# Mental Health in Settings with COVID-19 Positive Cases in the Spanish Population: The Protective Role of the Capacity to Adapt to Change

**DOI:** 10.3390/jcm11061497

**Published:** 2022-03-09

**Authors:** María del Mar Molero Jurado, María del Carmen Pérez-Fuentes, Iván Herrera-Peco, Nieves Fátima Oropesa Ruiz, Ana Belén Barragán Martín, África Martos Martínez, María del Mar Simón Márquez, José Jesús Gázquez Linares

**Affiliations:** 1 Department of Psychology, Faculty of Psychology, University of Almería, 04120 Almeria, Spain; mmj130@ual.es (M.d.M.M.J.); mpf421@ual.es (M.d.C.P.-F.); foropesa@ual.es (N.F.O.R.); abm410@ual.es (A.B.B.M.); msm112@ual.es (M.d.M.S.M.); jlinares@ual.es (J.J.G.L.); 2 Nursing Department, Health Sciences Collegue, Alfonso X El Sabio University, 28691 Madrid, Spain; iherrpec@uax.es; 3 Department of Psychology, Universidad Autónoma de Chile, Providencia 7500000, Chile

**Keywords:** adaptation to change, COVID-19, emotion, pandemic, health

## Abstract

Background: The capacity to adapt to change in complex, highly demanding situations, such as those derived from the COVID-19 pandemic, is essential in maintaining one’s mental health. This study analyzed the mental health of the Spanish population in settings with COVID-19 positive cases and the protective role of adaptation to change. Methods: The sample consisted of 1160 adult Spaniards aged 18 to 82 with a mean age of 38.29 (*SD* = 13.71). Data were collected by a CAWI (Computer Aided Web Interviewing) survey which included the General Health Questionnaire (GHQ-28), Adaptation to Change Questionnaire (ADAPTA-10), and an ad hoc questionnaire related to COVID-19 (perceived economic impact, COVID-19 positive diagnosis or not, and whether there was a positive case close to them). Results: The data revealed that the perceived economic impact showed a negative association between the emotional factor and the total score in adaptation to change. Lastly, the emotional factor in adaptation to change operated as a protector from the effect that a setting with COVID-19 positive cases exerts on mental health. Conclusions: Coping with COVID-19 requires strengthening the capacity for adaptation to changes generated in the setting, especially emotional, as it could contribute to improving the mental health of individuals, especially in those settings where they find and know a COVID-19 positive case.

## 1. Introduction

In view of the worldwide situation generated by the COVID-19 pandemic [1], it is essential to know how individuals adapt to the many changes caused by the virus in their everyday affairs and the effects of this capacity for adaptation on public mental health.

Capacity for adapting refers to functional modification of the individual’s psychological and behavioral responses to change. These changes must have beneficial results enabling them to successfully cope with events and daily demands [2]. Furthermore, the most contemporary approaches to health response focus on maintaining individual physical and mental wellbeing, and its corresponding repercussions on society [3]. The implication of the concept of health in the social setting is essential for referring to adequate integration of individuals in the society they pertain to [4].

The COVID-19 pandemic has affected individual physical health due to its severe symptoms [5,6]. However, it has also affected the mental dimension of health, since the disease itself, actions for its control, and access to information have modified usual individual and social behavior [7,8,9,10]. These changes in how one relates to the setting may cause individuals to become vulnerable [11], contributing to the appearance of psychological symptoms such as anxiety, irritability, fury, frustration, feelings of loneliness, fear, and intolerance to uncertainty [9,12,13,14,15,16,17,18,19].

Returning to the capacity for adaptation, this variable is considered a skill or disposition for change in behavior in the face of modifications in one’s activities, behaviors or social norms, and even changes in setting [20]. The capacity for adaptation is demonstrated when any change occurs, whether or not it has negative connotations for the individual.

In this adaptation, resilience may help overcome such adverse stressful situations [21]. Resilience may be defined as “bouncing back” from difficult experiences, so that individuals adapt to stressful changes, traumas, tragedies, or even settings or situations, while their usual social behaviors remain unaltered, that is, stable and functional [22,23]. It is therefore a protective mechanism against the pernicious effects of stress [24]. Some stressful situations that could require an individual’s capacity for resilience are death, natural disasters, or other catastrophic events, such as a pandemic [25], and adverse economic situations [26,27].

Not everyone has the same capacity for adaptation to change in their setting, since resilience is a phenomenon affected by a multitude of factors [22]. One factor influencing it is sex, as women adapt worse to stressful changes in their setting [28]. Another is age, as older age has been found to be associated with higher capacity for resilience, which, in turn, is associated with stronger and more resolute purpose in life [29,30]. Other factors related to resilience may be trained and improved, such as positive coping [31,32], and anxiety and stress reduction techniques [33] can improve the capacity for adaptation [22,34]. Training in coping strategies for stressful or catastrophic situations enables vulnerability and exhaustion to be reduced and a more precise and efficient response to instructions from authorities [35,36,37]. Management and coping therefore exert a fundamental role in adaptation to change.

Two main dimensions should be mentioned with regard to coping with stressful situations: (i) cognitive-behavioral and (ii) emotional [2].

The cognitive-behavioral dimension includes factors associated with control, management, and action taken to adapt to change, such as awareness, cognitive flexibility, and stress management. Likewise, among the determinants involved in the emotional dimension that influence the capacity for adapting to change are social support and tolerance to uncertainty, not omitting depression and anxiety [2,38].

In the emotional dimension of adaptation to change, depression is associated with functional alterations, which may include limitations on activity, reduced concentration, low energy or fatigue, and others [39].

It should also be mentioned that social support reinforces resilience or adaptability to stressful events, reducing their impact on the individual [20,40]. Social support has a structural dimension in the size of the individual’s contact network and the frequency of those contacts, but also a functional dimension that is related to empathy or support received [41]. People with a numerous and functional social support network can improve their resilience better and cope positively with stressful events, improving their quality of life [31], and can redefine a situation so it is no longer stressful [42].

In addition, tolerance to uncertainty may be defined as individuals’ cognitive and emotional responses to unknown situations [43]. It is worth mentioning that the situation associated with COVID-19, related to not having adequate, understandable information, generates uncertainty [44], which, in turn, is associated with the appearance of anxiety, stress, and emotional alterations [45,46]. In the light of these findings, managing emotions takes on considerable importance for adapting to situations that generate uncertainty. Therefore, it seems likely that those with better capacity for emotional management will also have better adaptive capacity [47].

### Purpose and Hypotheses

Based on the discussion above, this study analyzed the capacity for adaptation to stressful situations, such as the COVID-19 pandemic, with attention to sociodemographic variables, setting characteristics, and mental health problems. Previous studies have focused on knowing how the facility for overcoming adversity, which generates wellbeing in individuals, is affected by psychological distress caused by COVID-19 [48,49]. In our study, we wanted to know whether there are negative effects on mental health if there is an inadequate adaptive response. We wanted to find out the indicators and characteristics of individuals at high mental health risk, which in turn enables intervention before it derives in negative clinical situations.

**Figure 1:**
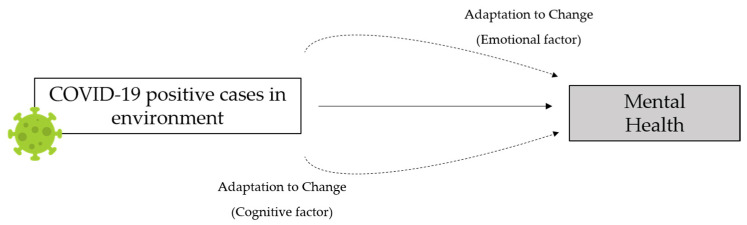
Hypothesized model of the mediating role of adaptation to change on the effect of COVID-19 on mental health.

The main objectives were to: (1) Identify the repercussion of situations derived from the COVID-19 pandemic, and (2) Study the mediating role of adaptation to change in settings where there are COVID-19 positive cases on mental health (Figure 1).

The following hypotheses were posed:

**Hypothesis** **1** **(H1).** *It was expected that there would be significant differences between men and women in the capacity for adaptation to change*.

**Hypothesis** **2** **(H2).** *The capacity for adapting to change would be positively correlated with age*.

**Hypothesis** **3** **(H3).** *The capacity for adapting to change would be negatively associated with the perceived economic impact of COVID-19*.

**Hypothesis** **4** **(H4).** *Adaptation to change would be negatively correlated to mental health problems*.

**Hypothesis** **5** **(H5).** *The emotional factor of adaptation to change would function as a mediator of the effect that a setting where there are COVID-19 positive cases has on one’s mental health*.

## 2. Materials and Methods

### 2.1. Participants

For the selection of the sample in this cross-sectional study, the inclusion criteria were being of legal age and having access to the internet. Those individuals who did not answer all the questions or did so randomly were discarded. Therefore, participation was 1688 adults, of whom 528 were discarded due to incomplete or random answers. The final sample was comprised of 1160 adult Spaniards aged 18 to 82 and a mean age of 38.29 (*SD* = 13.71).

Of the total sample, 30.1% (*n* = 349) were men and 69.9% (*n* = 811) were women, with a mean of age of 41.16 (*SD* = 14.13) and 37.05 (*SD* = 13.34), respectively. Of these, 47% (*n* = 545) did not have a stable partner and 53% (*n* = 615) did. Concerning education, 77% of the sample had a higher education (*n* = 893), and the rest were distributed between primary (3.5%, *n* = 41) and secondary (19.5%, *n* = 226) education.

They were asked if they had been diagnosed as COVID-19 positive (1.6% of the sample answered affirmatively, *n* = 19), and whether there any positive cases close to them, to which 31% (*n* = 360) said yes. Concerning the perceived economic impact from COVID-19, 53.1% (*n* = 616) of the participants said little or none, 24.1% (*n* = 281) somewhat, and the remaining 22.7% (*n* = 263) stated that it had affected them quite a lot or very much.

### 2.2. Instruments

An ad hoc questionnaire was drafted to collect participant sociodemographic data and matters related to circumstances having to do with COVID-19 (perceived economic impact, COVID-19 positive diagnosis or not, and whether there was a positive case close to them).

Adaptation to Change Questionnaire (ADAPTA-10) [2]. This instrument consists of 10 items answered on a five-point Likert-type scale (from “not at all” to “very much”). It provides a total score on ability to change, but also information on: (i) emotional factor, related to anguish and distress that may appear due to change, and (ii) cognitive-behavioral factor, related to the capacity for controlling, managing, and acting in different situations. Reliability was *ω* = 0.83 and GLB = 0.90 for the total score, *ω* = 0.85 and GLB = 0.87 for the emotional factor and *ω* = 0.75 and GLB = 0.78 for the cognitive-behavioral factor.

General Health Questionnaire (GHQ-28) [50], Spanish adaptation validated by Lobo et al. [51]. It consists of 28 items with four answer choices that provide information on the somatic symptoms, anxiety and insomnia, social dysfunction, and depression subscales. The Likert scale correction method was used, attributing answers cores from 0 to 3. In our case, instrument reliability was *ω* = 0.93 and GLB = 0.94 for the complete scale, and for each of the subscales: somatic symptoms (*ω* = 0.86; GLB = 0.89), anxiety/insomnia (*ω* = 0.90; GLB = 0.95), social dysfunction (*ω* = 0.81; GLB = 0.82), and depression (*ω* = 0.91; GLB = 0.94).

### 2.3. Procedure

Data were collected in a CAWI (Computer Aided Web Interviewing) survey by snowball sampling, from 1–12 May 2020. Participation was voluntary and, before starting to answer the questionnaire, essential information about the study was provided, as well as personal data management and processing matters. The participants gave their consent by marking a box designated for the purpose, which then gave them access to the questionnaire.

Control questions were distributed throughout the test to detect random or incongruent answers. This study was approved by the University of Almería Bioethics Committee (UALBIO2020/032).

### 2.4. Data Analysis

First, to find out whether there were any differences in capacity for adaptation to change, the independent samples *t*-test was applied, and the Cohen’s *d* [52] was estimated to quantify the effect size. In addition, to test the relationships between variables, Pearson’s coefficient correlation analyses were done.

As a strategy for identifying profiles by COVID-19 characteristics in the setting, a two-stage cluster analysis was performed. To determine whether there were any differences between COVID-19 clusters related to the mean scores on adaptation to change, an ANOVA with post hoc correction (Bonferroni) was calculated. For the size effect, the *η*^2^ and *ω*^2^ were estimated.

Finally, different mediation analyses were carried out, taking as the predictor (presence of a COVID-19 positive case nearby), as mediators the factors of adaptation to change and, as result variables, the health measure subscales (somatic symptoms, anxiety/insomnia, social dysfunction, and depression). JASP version 0.11.1 [53] based on lavaan software was used for this [54]. The bias-corrected percentile bootstrap confidence intervals method was used, as suggested by Biesanz, Falk, and Savalei [55]. Reliability was estimated with the McDonald’s Omega and the Greatest Lower Bound (GLB).

## 3. Results

This section may be divided by subheadings. It should provide a concise and precise description of the experimental results, their interpretation, as well as the experimental conclusions that can be drawn.

### 3.1. Adaptation to Change: Sociodemographic Variables and COVID-19 in the Setting

First, age was found to be positively correlated with the emotional factor (*r* = 0.18; *p* < 0.001; 95% CI 0.12, 0.23), the cognitive-behavioral factor (*r* = 0.17; *p* < 0.001; 95% CI 0.11, 0.22), and the total score on the adaptation to change scale (*r* = 0.21; *p* < 0.001; 95% CI 0.15, 0.26).

**Figure 2:**
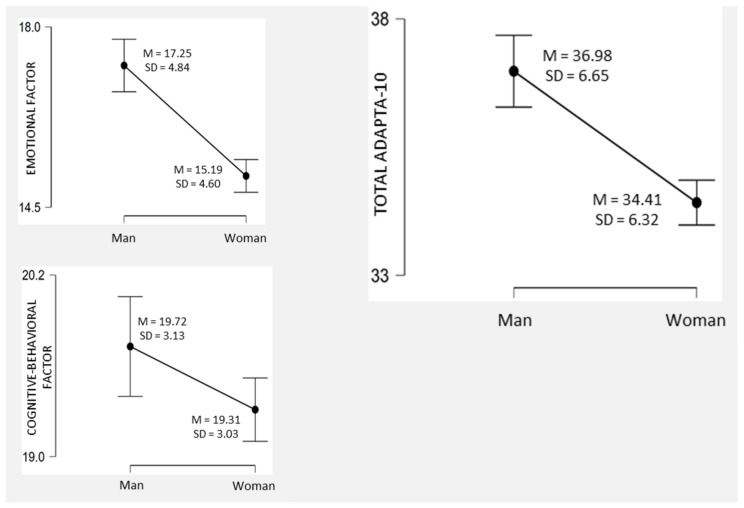
Adaptation to change by sex, descriptive plots.

Participant sex was found to have statistically significant differences in all the adaptation measures (Figure 2). Specifically, men had higher mean scores on the emotional factor (*t*_(1158)_ = 7.15; *p* < 0.001; *d* = 0.45), the cognitive-behavioral factor (*t*_(1158)_ = 2.12; *p* < 0.05; *d* = 0.13), and also the total adaptation scale (*t*_(1158)_ = 6.22; *p* < 0.001; *d* = 0.39).

From the perspective of the sentimental situation (partner/no partner) at the time data were collected, differences were found for the emotional factor (*t*_(1158)_ = −2.37; *p* < 0.01; *d* = −0.14), the cognitive-behavioral factor (*t*_(1158)_ = −4.30; *p* < 0.001; *d* = −0.25), and the total adaptation scale score (*t*_(1158)_ = −3.76; *p* < 0.001; *d* = −0.22), where those who had a partner scored higher (EM: *M* = 16.60, *SD* = 4.58; CB: *M* = 19.79, *SD* = 2.73; Total: *M* = 35.86, *SD* = 6.04) than those who did not (EM: *M* = 15.40, *SD* = 4.97; CB: *M* = 19.02, *SD* = 3.36; Total: *M* = 34.42, *SD* = 6.97).

The data on education did not back any significant association with the total score in adaptation to change (*r* = 0.02; *p* = 0.467), the emotional factors (*r* = 0.01; *p* = 0.802), or cognitive-behavioral factors (*r* = 0.03; *p* = 0.247).

From another perspective, based on the cluster analysis, the participants were classified by their answers to the questions: “Have you been diagnosed as COVID-19 positive?” and “Do you have or have you had someone COVID-19 positive close to you?”. This resulted in three clusters: Cluster 1 (C1), which was the most numerous (68.6%, *n* = 796), collected those who had not been diagnosed as COVID-19 positive nor had any positive case near them; Cluster 2 (C2) with 29.7% (*n* = 345) of the sample included those who had not been diagnosed positive but said there was some positive case near them; and, finally, Cluster 3 (C3), the smallest (1.6%, *n* = 19), contained those who had been diagnosed as COVID-19 positive and also had a case in their setting.

Table 1 shows the results found from the comparison of means in adaptation and the analysis of variance, by a COVID-19 cluster.

The mean scores differed by cluster for the emotional factor and total adaptation score. In the emotional factor, the post hoc tests showed that the significant differences found were specifically in the C1–C3 comparison, where C1 has a significantly higher mean score (c1 > c3_MD_ = 3.19; 95% CI 0.60–5.78). For the total adaptation score, the differences were in the c1–c3 (c1 > c3_MD_ = 4.43; 95% CI 0.88–7.98) and c2–c3 (c2 > c3_MD_ = 3.85; 95% CI 0.25–7.45) comparisons. Finally, regarding the perceived economic impact due to the COVID-19 pandemic, negative correlations were found with the emotional factor (*r* = −0.18; *p* < 0.001; 95% CI −0.24, −0.13), and with the total on adaptation to change (*r* = −0.14; *p* < 0.001; 95% CI −0.20, −0.08). No significant association was found between the perceived economic impact and the cognitive-behavioral factor (*r* = −0.01; *p* = 0.606).

### 3.2. Adaptation to Change and Health

Table 2 shows the correlation matrix between the factors and total score on the ADAPTA-10 adaptation to change questionnaire and the different GHQ-28 subscales. Both factors (emotional and cognitive-behavioral) and total score in adaptation showed negative correlations with the presence of health problems: somatic symptoms, anxiety/insomnia, social dysfunction, and depression.

**Figure 3:**
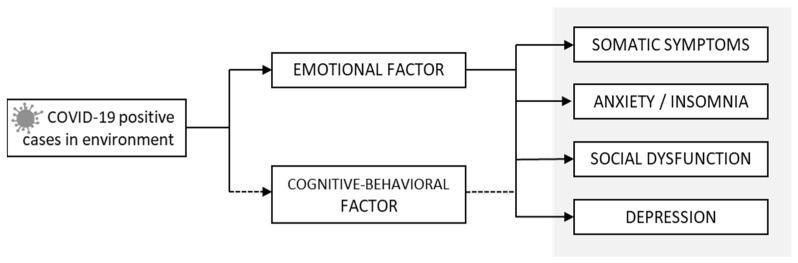
Hypothesized mediation model.

A mediation analysis was computed (Figure 3) to check the mediating role of the capacity for adapting to change, and, in both cases, the predictor was presence of some close COVID-19 positive case, and the GHQ-28 dimensions were the output variables.

As shown in Table 3, there was a significant direct effect of close COVID-19 positive cases on the presence of somatic symptoms. Insofar as the indirect effects, the emotional factor of adaptation to change was a significant mediator in the relationship between close COVID-19 positive cases and the four health subscales. The total effects were significant for the output variables: somatic symptoms, anxiety/insomnia, social dysfunction, and depression.

## 4. Discussion

This study analyzed the emotional and cognitive dimensions that make up the capacity for adapting to change during a threat such as COVID-19 and its possible relationship to perceived health.

First, differences were found in adaptation to change by sex. Men had higher scores than women, both in the emotional and cognitive-behavioral factors and in general adaptation to change [2]. These data are in agreement with what has been reported previously in the literature, where men have a better capacity for adapting to adverse events and situations [28], and a lower level of vulnerability to threats such as COVID-19 [8].

With regard to the second hypothesis of this study, the results showed an effect of age on the capacity for adapting to change. Older individuals had a better score on emotional, cognitive-behavioral, and general adaptation, indicating better capacity for adapting to the stressful situation that COVID-19 represents. These data were associated with older individuals who had more experience, higher resilience, and coherence in their coping structures in unforeseen events [29,30]. Moreover, older individuals have a wider, more consolidated contact network [41], which could enable them to adapt better to certain adverse events [20,31,42], such as COVID-19 and the actions taken for its control [6,7,9].

Analysis of the economic impact perceived by the population as attributable to COVID-19 showed a relationship between this concern and emotional factors and general adaptation to the changing situation generated by the disease, while no relationship was found with the cognitive-behavioral factors. Loss of one’s job or lower purchasing power diminish the standard of living and satisfaction [27], causing alterations in one’s behavioral level and even less ability to tolerate change [26]. Loss of economic resources is also associated with the generation of a feeling of loneliness and being abandoned [9], anxiety, irritability, and depression [13,16,17].

It was also observed that those who had a stable partner not only had higher scores in total adaptation, but also higher emotional and cognitive levels. Having a stable couple positively influences the capacity for adaptation to adverse events, which agrees with what has been described by other authors who have indicated that maintaining a stable partner increases wellbeing [27] and provides emotional and social support [56] even in catastrophic events.

However, the level of education of the participants was not related to adaptive capacity in events such as the COVID-19 pandemic, a result which does not agree with those previously published, where the level of education was important to resilience and preparation for resisting catastrophic events, finding that those who had a higher education were better prepared than those with a lower level of education [35].

Finally, the appearance of COVID-19 positive cases in one’s close setting was found to be associated with a lower capacity for adapting to the changes generated and the appearance of more prevalence of health disorders. Close COVID-19 positive cases cause a stressful situation [7,9], which could lead to uncertainty and feeling symptoms compatible with COVID-19, which could then cause alterations in behavior [21,22,23], anxiety [19], and depression because the perceived danger to oneself or one’s family cannot be managed [39].

Concerning the relationship between the capacity for adapting to change and mental health, a significant negative relationship was found between adaptation to change, the emotional and cognitive factors, and health. Thus, individuals who had a greater capacity for adapting to change, both in its cognitive and emotional facets, in general, scored lower in all the symptoms: somatic, anxiety/insomnia, depression, and social dysfunction [50,51]. Other studies have found that capacity for emotional management is related to better capacity for adapting to changing situations [33,46,47], while, on the contrary, depressive states that could arise in situations of extreme stress have been related negatively to successful coping or better capacity for adapting to new situations [24], and worse psychological wellbeing [48,49]. The results of other studies also insist on the importance of being able to depend on a wide social support network to cope more successfully with stressful situations and reinforce the capacity for individual resistance [20,31,42]. In brief, a better capacity for managing emotions and improvement in capacity for adapting to situations that generate uncertainty [22,34] lead to better perceived health.

This study also made an in-depth analysis of the protective effect that capacity for adapting to change exerts on the relationship between settings with a COVID-19 positive case and mental health. The importance of the protective role exerted by the emotional factor of capacity for change was shown, confirming our last research hypothesis. These findings reinforce the need to learn to manage emotions, especially anxiety and distress, which can appear in response to change, to contribute to wellbeing, recovery, and general health in settings with COVID-19 positive cases.

### 4.1. Practical Implications

Our results show that the mental health of the population may be affected by a setting with positive COVID-19 cases. In this scenario, effective emotional coping is fundamental to prevent psychological symptoms associated with a pandemic. We also found data suggesting that it is hardest for young people and women to adapt to changes derived from surroundings with positive COVID-19 cases. Information on such risk profiles provides an advantage for making the right decisions on preventive action for improving wellbeing and mental health.

Any initiative directed at improving the capacity for adaptation to change has positive consequences for protecting public mental health. Therefore, the data derived from this study have important practical implications for decision-making on the design of interventions for the general public. In this line of action, agents of social change (community social services, healthcare personnel, educators, and so forth) could benefit from these results. Other factors involved in the process of adapting to change, such as resilience or emotional intelligence, should be studied in depth and included in specific intervention programs, with the appropriate adaptations to the sociocultural context and populational characteristics in each case.

### 4.2. Limitations and Future Research

Although some of our findings on psychological coping with COVID-19 may have positive repercussions, this study also had some limitations, mainly related to the study design, which was cross-sectional. These data should be completed with longitudinal studies that enable causal relationships between the variables studied to be established. In addition, although the presence of close positive cases was linked to worse mental health, it is true that not all COVID-19 patients have the same symptoms or the same severity. Therefore, future research should employ mixed methodologies that would enable the psychological effects of close family and friends of COVID patients to be studied based on the severity of the effects of the virus, thereby establishing effective measures for reducing distress in both the patients themselves and in their close circle. Finally, we should mention that this study only considered primary sociodemographic characteristics (such as sex or age). Other secondary sociodemographic characteristics, such as dependents (children or invalid), religious beliefs, or being vaccinated, could exert a relevant role in the capacity for adaptation and mental symptoms. Therefore, future studies should employ measures of such characteristics. This way, a more precise map could be drawn of the vulnerability of the population to nonphysical effects of COVID-19.

## 5. Conclusions

The results of this study advance knowledge of the capacity for adaptation to changes generated by COVID-19 in the Spanish population. As discussed above, the presence of close positive cases has an impact on an increase in psychological symptoms (such as depression, anxiety and insomnia, social dysfunction, and somatization) through the emotional response, which occurs while adapting emotionally to new situations. Thus, improvement in the capacity for emotional adaptation to change can generate benefits in mental health of individuals with family members or close friends infected by COVID-19. This is especially important because, unlike what was popularly believed in the beginning, it now seems that COVID-19 is not going to end, but, on the contrary, tends to become an endemic disease spreading to more and more people.

Therefore, as our findings show that the emotional facet of adaptation to change is the most relevant ingredient in psychological wellbeing of the population with close positive cases, we firmly believe in the need to create prevention and intervention measures that improve it. Thus, healthcare authorities should focus on designing psychosocial programs that work on the emotional intelligence of the population to improve coping with COVID-19 and the threatening or uncertain situations it generates. We recommend preventive action to reduce the presence of psychological symptoms in the Spanish population through programs for improving emotional competence. These could be implemented in schools and training centers, workplaces, or community activity centers, which would increase the possibilities of covering a wide spectrum of the population. Nevertheless, based on these findings, the accent should be placed on young adults and women, so women’s centers and higher vocational training schools and universities are postulated as two locations of special interest for these programs.

Finally, we emphasize that governmental healthcare promotion should be directed at improving public health, not forgetting that mental wellbeing is an indispensable component of this integral attention. Attending to psychoemotional needs is urgent if an effective response to mental health problems, including both their clinical manifestations and early detection, are to be achieved.

## Figures and Tables

**Table 1:** Adaptation to change by a COVID-19 cluster, descriptive statistics, and ANOVA.

	Emotional Factor			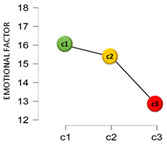
	*n*	*M*	*SD*	
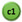 Clúster 1	796	15.98	4.77	
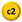 Clúster 2	345	15.38	4.74	
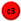 Clúster 3	19	12.78	4.52	
*F* = 5.67; *p* < 0.01 (*η*² = 0.010, *ω*² = 0.008)				
	Cognitive-Behavioral Factor			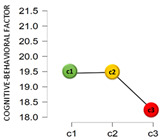
	*n*	*M*	*SD*	
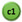 Clúster 1	796	19.44	3.15	
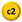 Clúster 2	345	19.47	2.83	
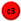 Clúster 3	19	18.21	3.32	
*F* = 1.55; *p* = 0.212				
	Total ADAPTA-10			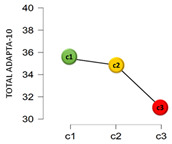
	*n*	*M*	*SD*	
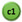 Clúster 1	796	35.43	6.56	
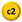 Clúster 2	345	34.85	6.36	
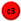 Clúster 3	19	31.00	6.80	
*F* = 4.93; *p* < 0.01 (*η^2^* = 0.008, *ω*^2^ = 0.007)				

**Table 2:** Adaptation to change and health. Pearson correlations.

		GHQ-SS		GHQ-AI		GHQ-SD		GHQ-D	
Emotional factor	Pearson’s *r*	−0.617	***	−0.792	***	−0.491	***	−0.551	***
	*p*-value	<0.001		<0.001		<0.001		<0.001	
	Upper 95% CI	−0.580		−0.770		−0.446		−0.509	
	Lower 95% CI	−0.651		−0.813		−0.534		−0.590	
Cognitive-Behavioral factor	Pearson’s *r*	−0.261	***	−0.311	***	−0.330	***	−0.391	***
	*p*-value	<0.001		<0.001		<0.001		<0.001	
	Upper 95% CI	−0.206		−0.258		−0.278		−0.341	
	Lower 95% CI	−0.314		−0.362		−0.381		−0.439	
Total ADAPTA-10	Pearson’s *r*	−0.574	***	−0.725	***	−0.515	***	−0.587	***
	*p*-value	<0.001		<0.001		<0.001		<0.001	
	Upper 95% CI	−0.534		−0.697		−0.471		−0.548	
	Lower 95% CI	−0.611		−0.752		−0.556		−0.623	

Note. GHQ-SS = Somatic symptoms, GHQ-AI = Anxiety/insomnia, GHQ-SD = Social dysfunction, GHQ-D = Depression. *** *p* < 0.001.

**Table 3:** Direct, indirect, and total effects.

**Direct Effects**						**95% CI**	
		**Estimate**	**Std. Error**	**z-Value**	***p***	**Lower**	**Upper**
COVID-19 positive cases in environment	→ GHQ-SS	0.228	0.050	4.611	<0.001	0.120	0.330
	→ GHQ-AI	0.068	0.039	1.744	0.081	−0.011	0.139
	→ GHQ-SD	−0.001	0.054	−0.019	0.984	−0.106	0.106
	→ GHQ-D	0.061	0.051	1.187	0.235	−0.034	0.179
**Indirect Effects**						**95% CI**	
		**Estimate**	**Std. Error**	**z-Value**	***p***	**Lower**	**Upper**
COVID-19 positive cases in environment	→ EM → GHQ-SS	0.089	0.038	2.375	0.018	0.018	0.165
	→ CB → GHQ-SS	5.229 × 10^−4^	0.003	0.166	0.868	−0.006	0.009
	→ EM → GHQ-AI	0.118	0.049	2.383	0.017	0.025	0.215
	→ CB → GHQ-AI	3.567 × 10^−4^	0.002	0.166	0.868	−0.004	0.007
	→ EM → GHQ-SD	0.065	0.027	2.361	0.018	0.013	0.120
	→ CB → GHQ-SD	0.002	0.011	0.166	0.868	−0.020	0.024
	→ EM → GHQ-D	0.071	0.030	2.367	0.018	0.014	0.132
	→ CB → GHQ-D	0.002	0.014	0.166	0.868	−0.025	0.029
**Total Effects**						**95% CI**	
		**Estimate**	**Std. Error**	**z-Value**	***p***	**Lower**	**Upper**
COVID-19 positive cases in environment	→ GHQ-SS	0.318	0.063	5.073	<0.001	0.178	0.442
	→ GHQ-AI	0.185	0.063	2.934	0.003	0.063	0.297
	→ GHQ-SD	0.065	0.063	1.033	0.302	−0.057	0.193
	→ GHQ-D	0.134	0.063	2.120	0.034	0.002	0.260

Note. EM = Emotional factor, CB = Cognitive-Behavioral factor, GHQ-SS = Somatic symptoms, GHQ-AI = Anxiety/insomnia, GHQ-SD = Social dysfunction, GHQ-D = Depression. (Note. Delta method standard errors, bias-corrected percentile bootstrap confidence intervals).

## Data Availability

The data that support the findings of this study are available from the corresponding author upon reasonable request.
